# 12-Fold symmetry of the putative portal protein from the *Thermus thermophilus* bacteriophage G20C determined by X-ray analysis

**DOI:** 10.1107/S174430911302486X

**Published:** 2013-10-17

**Authors:** Lowri S. Williams, Vladimir M. Levdikov, Leonid Minakhin, Konstantin Severinov, Alfred A. Antson

**Affiliations:** a York Structural Biology Laboratory, Department of Chemistry, University of York, York YO10 5DD, England; b Department of Molecular Biology and Biochemistry, Rutgers, The State University of New Jersey, Piscataway, NJ 08854, USA; c Waksman Institute for Microbiology, Rutgers, The State University of New Jersey, Piscataway, NJ 08854, USA; d St Petersburg State Polytechnical University, St Petersburg 195251, Russian Federation

**Keywords:** putative portal protein, *Thermus thermophilus*, bacteriophage G20C

## Abstract

In tailed bacteriophages and several animal viruses, the portal protein forms the gateway through which viral DNA is translocated into the head structure during viral particle assembly. In the mature virion the portal protein exists as a dodecamer, while recombinant portal proteins from several phages, including SPP1 and CNPH82, have been shown to form 13-subunit assemblies. A putative portal protein from the thermostable bacteriophage G20C has been cloned, overexpressed and purified. Crystals of the protein diffracted to 2.1 Å resolution and belonged to space group *P*42_1_2, with unit-cell parameters *a* = *b* = 155.3, *c* = 115.4 Å. The unit-cell content and self-rotation function calculations indicate that the protein forms a circular 12-subunit assembly.

## Abstract 2

Crystal data on a putative portal protein from the thermostable bacteriophage G20C indicate that it forms a 12-subunit assembly.

## 1. Introduction

During the assembly of tailed dsDNA bacteriophages, a copy of the viral genome is packaged into a preformed protein shell known as a procapsid. The portal protein, a circular oligomer, is embedded into one of the vertices of the icosahedral procapsid (Rao & Feiss, 2008 ▶; Casjens, 2011 ▶). The portal protein primarily functions to connect other viral proteins to the procapsid and as the gateway through which DNA is translocated into the procapsid and out of the mature capsid. Typically, following replication of the viral genome, a complex comprising the small and large terminase proteins and the viral DNA binds to the portal protein to form an ATPase-driven DNA-translocating motor. The motor drives the viral DNA through the portal protein and into the procapsid, where the DNA is packaged to near-crystalline density. Following DNA packaging and dissociation of the terminase complex, the portal protein binds components of the tail structure to complete the viral assembly process. On infection of a host cell, DNA leaves the mature capsid through the portal protein and tail structure.

In functional mature viral particles and following isolation in complex with tail proteins, the portal proteins from several bacteriophages, such as SPP1 and T3, have consistently been identified as dodecameric rings with 12-fold rotational symmetry (Lurz *et al.*, 2001 ▶; Donate *et al.*, 1988 ▶; Rao & Feiss, 2008 ▶). These results strongly suggest that the biologically relevant oligomeric state of these portal proteins is a dodecamer. Following heterologous expression, however, viral portal proteins have been found to display 11-fold, 12-fold, 13-fold or 14-fold symmetry (Trus *et al.*, 2004 ▶; Rao & Feiss, 2008 ▶). For example, the SPP1 and CNPH82 portal proteins exhibit 13-fold symmetry following heterologous expression in *Escherichia coli* (Lebedev *et al.*, 2007 ▶; Lurz *et al.*, 2001 ▶; Luan *et al.*, 2012 ▶). This suggests that dodecamers may be selected for, or their assembly may be promoted, during the native oligomerization process and that the dodecameric arrangement of the portal protein is important for its function (Rao & Feiss, 2008 ▶; Lurz *et al.*, 2001 ▶).

Despite the portal proteins from different tailed bacteriophages varying significantly in both amino-acid sequence and molecular mass, they all assemble into circular homo-oligomers that have a turbine-like shape and contain a central channel for DNA translocation (Luan *et al.*, 2012 ▶; Orlova *et al.*, 1999 ▶). The X-ray structures of several bacteriophage portal proteins subsequently revealed additional common structural features and shed light on the mode of action of the portal proteins (Lebedev *et al.*, 2007 ▶; Olia *et al.*, 2011 ▶; Simpson *et al.*, 2001 ▶). Common features of bacteriophage portal proteins include the presence of negatively charged residues lining the central channel, which would favour translocation of negatively charged DNA through the channel, and several conserved structural motifs. One such motif is formed by three α-helices comprising two tunnel helices, a perpendicular long helix and the tunnel loop. Another prominent conserved feature is a ‘clip’ structure at the base of the portal protein (Lebedev *et al.*, 2007 ▶; Rao & Feiss, 2008 ▶).

In this paper, we report the expression and purification of a putative portal protein from the *Thermus thermophilus* bacterio­phage G20C, a close relative of bacteriophages P23-45 and P74-26 (Minakhin *et al.*, 2008 ▶). Initial trials with the wild-type protein comprising 448 residues resulted in the production of an insoluble protein, but this was remedied by the use of N- and C-terminal truncations. A soluble and stable protein construct was crystallized and its symmetry was deduced from the crystal data. This provides a route to characterize the structure and mechanism of action of this protein.

## 2. Materials and methods

### 2.1. Cloning, expression and purification

In bacteriophage genomes, the gene encoding the portal protein is usually positioned directly after the gene encoding the large terminase. The gene encoding the G20C large terminase containing the classical Walker motifs was annotated by sequence homology to the large terminase from bacteriophage P23-45. Based on the genomic context and size of the gene, and the predicted secondary structure of the gene product, the gene directly following the large terminase is likely to encode the portal protein of G20C. The gene corresponds to the ORF P23p86 (UniProtKB/TrEMBL A7XXB9) in the closely related phage P23-45 (Minakhin *et al.*, 2008 ▶).

Forward and reverse primers containing the *Nde*I and *Bam*HI restriction-site sequences, respectively, were designed to incorporate a hexahistidine tag at the N-terminus of the DNA sequence encoding the truncated putative portal protein (Ser21–Asp438). The amplified segment was cloned into the pET-22a vector (Novagen). Sequencing and alignment were performed to confirm the sequence of the insert.

The truncated putative G20C portal protein bearing an N-terminal hexahistidine tag was overexpressed in *E. coli* strain B834. Cells were grown in Luria–Bertani medium with 100 µg ml^−1^ ampicillin at 310 K to mid-log phase (optical density of approximately 0.6 at 600 nm). Expression of the portal protein was induced by the addition of 0.1 m*M* IPTG followed by incubation at 289 K for 20 h. The cell pellet was lysed by sonication at 277 K in lysis buffer consisting of 50 m*M* HEPES pH 7.5, 1 *M* NaCl, 5 m*M* imidazole and one cOmplete EDTA-free Protease-Inhibitor Cocktail tablet per 25 ml of solution (Roche). Nickel-affinity chromatography was performed on a 5 ml HisTrap Chelating HP column (GE Healthcare). The binding and elution buffers consisted of 50 m*M* HEPES, 1 *M* NaCl pH 7.5 with 5 and 500 m*M* imidazole, respectively. The protein was concentrated to approximately 10 mg ml^−1^ using a 30 kDa Ultra centrifugal filter (Amicon). The protein sample was purified further on a Superose 6 size-exclusion column (GE Life Sciences) in buffer consisting of 10 m*M* HEPES, 1 *M* NaCl pH 7.5. Purity was assigned by denaturing PAGE. The molecular mass of the purified sample was confirmed by matrix-assisted laser desorption/ionization mass spectrometry (MALDI–MS).

### 2.2. Crystallization

The protein was concentrated to approximately 10 mg ml^−1^ using a 30 kDa Ultra centrifugal filter (Amicon) in 10 m*M* HEPES, 1 *M* NaCl pH 7.5. Crystallization conditions were evaluated using standard commercial screens [Index and MPD (Hampton Research) and PACT (Molecular Dimensions)]. Drops composed of 150 nl purified protein solution and 150 nl reservoir solution were dispensed by a Mosquito nanolitre pipetting robot (TTP LabTech) and equilibrated against 60 µl reservoir solution. The best crystal was obtained from the MPD screen with a reservoir consisting of 0.2 *M* magnesium chloride, 40%(*v*/*v*) MPD.

### 2.3. X-ray data collection and processing

X-ray data were collected from a single cryocooled crystal on the I02 beamline at the Diamond Light Source, UK equipped with a Dectris Pilatus detector. Data were collected at a wavelength of 0.9795 Å with a crystal-to-detector distance of 321.2 mm, a 0.2° crystal rotation per image and a total crystal rotation range of 180°. The data were processed with *XDS* using the *xia*2 program (Kabsch, 2010 ▶; Winter *et al.*, 2013 ▶). The self-rotation function was calculated using *MOLREP* (Vagin & Teplyakov, 2010 ▶) with a resolution range of 51–2.89 Å and a radius of integration of 52.5 Å.

## 3. Results and discussion

### 3.1. Cloning, expression and purification

The truncated construct comprising an N-terminal methionine-hexahistidine tag and the Ser21–Asp438 protein segment contains 425 amino acids with a theoretical molecular mass of 47.2 kDa. This protein construct was cloned and overexpressed in *E. coli* B834 cells. Homogeneous protein was obtained after Ni-affinity and size-exclusion chromatography. The molecular weight of the purified protein measured by MALDI–MS was 47.022 kDa, which is in good agreement with the theoretical value of 47.027 kDa for the protein construct lacking the initial methionine residue.

### 3.2. Crystallization and crystal data

The best crystal was obtained using ∼10 mg ml^−1^ protein solution in 10 m*M* HEPES, 1 *M* NaCl pH 7.5 and a reservoir consisting of 40% MPD, 0.2 *M* magnesium chloride. The crystal belonged to the tetragonal space group *P*42_1_2, with unit-cell parameters *a* = *b* = 155.3, *c* = 115.4 Å. A complete X-ray data set to a resolution of 2.1 Å was collected on the I02 beamline at the Diamond Light Source (Table 1 ▶).

### 3.3. X-ray data analysis

**Figure 1:**
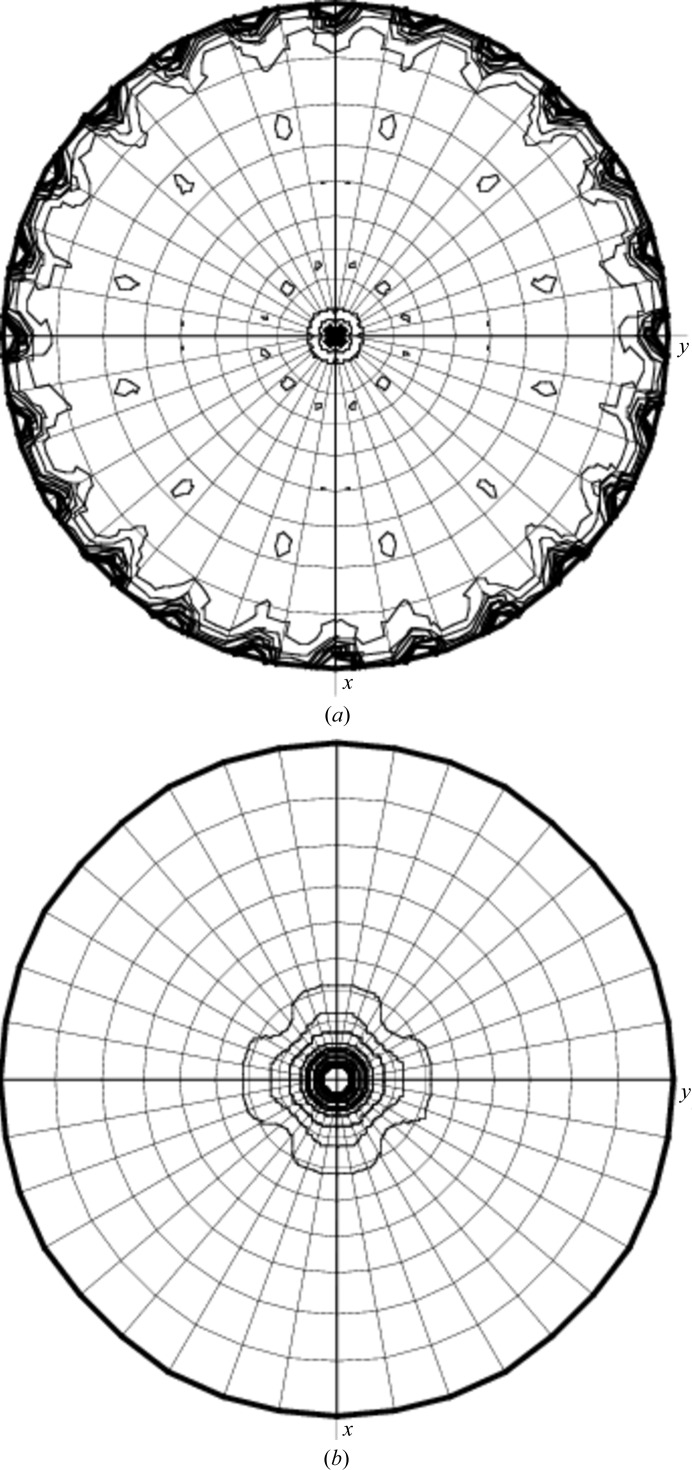
X-ray analysis. Stereographic projections of the self-rotation function: (*a*) κ = 180°, (*b*) κ = 30°.

The self-rotation function (Crowther, 1972 ▶) was calculated to deduce the internal symmetry of the portal protein. Peaks appearing in the κ = 180° section are related by a 30° rotation around the axis coinciding with the crystallographic fourfold axis (Fig. 1 ▶ *a*). Consistent with the presence of the 12-fold rotation symmetry, there is a peak in the κ = 30° section (Fig. 1 ▶ *b*) which is approximately 40% higher than the peaks in the κ = 32.7° (*i.e.* 360°/11) and κ = 27.7° (*i.e.* 360°/13) sections. Three subunits in the asymmetric unit correspond to a specific volume of 2.46 Å^3^ Da^−1^ and a solvent content of 50% (Matthews, 1968 ▶). The crystallographic fourfold symmetry generates a 12-subunit oligomer.

## 4. Conclusions

Following heterologous expression, the putative portal protein from bacteriophage G20C has been purified and crystallized. Analysis of the X-ray data collected to 2.1 Å resolution indicates that the protein forms a 12-subunit circular assembly. The genomic context, the size and the oligomeric state of the protein are consistent with it being a portal protein. Determination of the structure of this putative portal protein by molecular replacement is not possible owing to a complete lack of sequence similarity to portal proteins for which the three-dimensional structure is available. The next stage of this project will focus on experimental phasing.

## Figures and Tables

**Table 1:** X-ray data statistics Values in parentheses are for the outermost resolution shell.

X-ray source	I02, DLS
Wavelength (Å)	0.97950
Temperature (K)	100
Space group	*P*42_1_2
Unit-cell parameters (Å)	*a* = *b* = 155.3, *c* = 115.4
Resolution range (Å)	51.8–2.1 (2.15–2.10)
No. of unique reflections	82518 (6019)
*R*_merge_ † (%)	9.1 (75.8)
*R*_meas_ ‡ (%)	9.5 (86.3)
CC_1/2_ §	99.9 (78.4)
Average *I*/σ(*I*)	24.1 (4.2)
Completeness (%)	100 (100)
Multiplicity	13.1 (13.5)
Wilson *B* factor (Å^2^)	40.8

† *R*_merge_ = 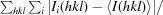 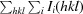, where *I_i_*(*hkl*) is the intensity of the *i*th measurement of a reflection with indices *hkl* and 〈*I*(*hkl*)〉 is the statistically weighted average reflection intensity.

‡ *R*_meas_ is the redundancy-independent *R* factor (Diederichs & Karplus, 1997 ▶).

§ CC_1/2_ is the percentage correlation between intensities from random half data sets (Karplus & Diederichs, 2012 ▶).
